# A Bayesian Spatial Model to Predict Disease Status Using Imaging Data From Various Modalities

**DOI:** 10.3389/fnins.2018.00184

**Published:** 2018-03-26

**Authors:** Wenqiong Xue, F. DuBois Bowman, Jian Kang

**Affiliations:** ^1^Boehringer Ingelheim Pharmaceuticals Inc., Ridgefield, CT, United States; ^2^Department of Biostatistics, The Mailman School of Public Health, Columbia University, New York, NY, United States; ^3^Department of Biostatistics, School of Public Health, University of Michigan, Ann Arbor, MI, United States

**Keywords:** Bayesian spatial model, prediction, MCMC, posterior predictive probability, importance sampling, Parkinson's disease

## Abstract

Relating disease status to imaging data stands to increase the clinical significance of neuroimaging studies. Many neurological and psychiatric disorders involve complex, systems-level alterations that manifest in functional and structural properties of the brain and possibly other clinical and biologic measures. We propose a Bayesian hierarchical model to predict disease status, which is able to incorporate information from both functional and structural brain imaging scans. We consider a two-stage whole brain parcellation, partitioning the brain into 282 subregions, and our model accounts for correlations between voxels from different brain regions defined by the parcellations. Our approach models the imaging data and uses posterior predictive probabilities to perform prediction. The estimates of our model parameters are based on samples drawn from the joint posterior distribution using Markov Chain Monte Carlo (MCMC) methods. We evaluate our method by examining the prediction accuracy rates based on leave-one-out cross validation, and we employ an importance sampling strategy to reduce the computation time. We conduct both whole-brain and voxel-level prediction and identify the brain regions that are highly associated with the disease based on the voxel-level prediction results. We apply our model to multimodal brain imaging data from a study of Parkinson's disease. We achieve extremely high accuracy, in general, and our model identifies key regions contributing to accurate prediction including caudate, putamen, and fusiform gyrus as well as several sensory system regions.

## 1. Introduction

Functional and structural neuroimaging play important roles in understanding the neurological basis for major psychiatric and neurological disorders such as Parkinson's disease (PD), schizophrenia, depression, and Alzheimer's diseases. There is emerging interest in using imaging and other clinical data to forecast or blindly classify subjects into subgroups, for example, defined by disease status or more refined diagnostic categories. Classification or prediction of disease status based on imaging data remains an active area of research and holds promise for making a significant clinical impact. Prediction models may have a range of applications and be beneficial for clinical diagnosis, determining antecedents to a standard diagnosis, forecasting prognosis, and revealing the underlying neural basis of disease, thus informing the development of future treatments.

We use data from a study of PD as a motivating example for our proposed methods (see section 2). Neuroimaging has revealed various functional and structural alterations associated with PD. There have been reports of cortical cortical thinning in PD patients determined from T1-MRI scans (Lee et al., 2013; Zarei et al., 2013; Zhang et al., 2015), decreased fractional anisotropy in the substantia nigra revealed by diffusion tensor imaging (DTI) (Vaillancourt et al., 2009), and functional connectivity, structural connectivity, and volumetric PD-related changes revealed by a multimodal imaging analysis (Bowman et al., 2016). These and other related studies suggest the utility of imaging data in revealing neuropathophysiology related the loss of dopamine producing neurons in PD and prompt the need for new methods to accommodate high-dimensional multimodal data.

Regularization and variable selection methods such as the least absolute shrinkage and selection operator (LASSO) (Tibshirani, 1996) and elastic-net (Zou and Hastie, 2005) as well as support vector classifiers are commonly used to predict a single scalar-valued outcome from high-dimensional data. Support vector classifiers, which arise from support vector machines (SVM), classify the data by constructing an optimal separating hyperplane in a high dimensional space to which the data are mapped (Cortes and Vapnik, 1995). Gaussian process (GP) models provide an alternative approach, which finds the posterior function that is closest to the training data based on Bayesian theory (Marquand et al., 2010). Ham and Kwak (2012) propose a boosted-principal component analysis (PCA) algorithm for binary classification problems that combines feature selection and classification.

Several methods have been proposed to predict follow-up imaging scans from baseline scans (Guo et al., 2008; Derado et al., 2013). Guo et al. (2008) propose a Bayesian hierarchical model for functional magnetic resonance imaging (fMRI) and positron emission tomography (PET) data; Derado et al. (2013) extends the model by introducing both spatial correlations between voxels and temporal correlations between baseline and follow-up functional imaging scans. For structural data, Stonnington et al. (2010) propose a relevance vector regression (RVR) model to predict the clinical scores using MRI T1 weighted scans.

Predicting disease status utilizes a potentially massive number of independent variables that exhibit unknown patterns of correlation. The prediction and classification models described above do not estimate the spatial correlations in imaging scans or capture the associations between different imaging modalities. We build on ideas of spatial modeling for correlated imaging data for our prediction framework. Specifically, we propose a novel Bayesian hierarchical model to predict disease status using imaging scans of different modalities in both gray and white matter to reflect the functional as well as the structural properties of the brain. We consider a two-level brain parcellation, dividing the brain into defined regions as well as subregions within regions, and assume different spatial correlation structures between voxels within a subregion, within a region, and in different regions. We perform Markov Chain Monte Carlo (MCMC) estimations via Gibbs sampling. The predictions for disease status are conducted based on the predictive posterior probabilities. Both whole-brain and voxel-level predictions are performed using leave-one-out cross validation (LOOCV). Also, we conduct feature selection to identify the regions that are associated with the disease based on the voxel-level prediction results. We apply our approach to a PD study and conduct simulation studies to evaluate its performance.

## 2. Parkinson's disease data

This research qualifies as Research of Existing Data, Records, Specimens [Basic Exempt Criteira 45 CFR 46.101(b)(4)], and has been deemed Not Human Subjects Research (HS Code 10 in IPMAC II as reference in the manual chapter 7410) by NIH and Columbia University Medical Center Institutional Review Board (Protocol: IRB-AAAO0062).

The data were originally collected at Emory University (P50-NS071669) and were supplied to Columbia with all subjects' records de-identied. Written and informed consent was obtained from all research participants at the time of data collection.

A total of 20 subjects, 11 of which are diagnosed as early to moderate PD patients and the rest are healthy controls, are included in the study. The average age is 66 (*s.d*. = 11) years, and 12 of the subjects are males. The mean duration of disease was 8.4 years (*s.d*. = 3.3). The average height is 175 cm and the average weight is 79 kg. Resting-state fMRI scans, and T1-weighted MRI scans, and diffusion tensor imaging (DTI) scans are obtained.

A Siemens Trio Tim 3T MRI scanner was used to capture all the imaging scans. MPRAGE was used to acquire the structural T1 scans (*TR* = 2,600 ms, *TE* = 3 ms, 192 sagittal slices at 1 mm; 256 × 232 1 mm isotropic pixels). The resting-state fMRI scans were acquired using echo planar imaging (EPI) (*TR* = 3,000 ms, *TE* = 30 ms, 48 axial slices at 3 mm, 128 × 128 2 mm isotropic pixels) for each subject. DTI data were captured using a biphase approach with consecutive left-to-right and right-to-left phase scans. The subjects followed a DTI protocol (*TR* = 8,700 ms, *TE* = 94 ms, 64 axial slices at 2 mm, 128 × 128 2 mm isotropic pixels) comprised of 64 directions (*B* = 1,000 s/mm2), with three leading and three trailing B0 scans.

We extract voxel-level information from these three imaging modalities, including fractional amplitude of low-frequency fluctuation (fALFF) from resting-state fMRI scans, voxel based morphometry (VBM) from T1-weighted MRI scans, and fractional anisotropy (FA) from DTI scans. fALFF reflects the amplitude of spontaneous blood-oxygen-level-dependent (BOLD) signal fluctuations of each voxel. VBM measures the localized gray matter volume changes in each voxel after spatially normalizing all the images to a standard space, and extracting gray matter from the normalized images (Ashburner and Friston, 2000). FA has a single value for each voxel, measuring the difference in directions along different axes of the random motion of water molecules in the brain, which reflects the physical orientation of white-matter fibers at that location. In summary, fALFF provides functional information, while FA and VBM describe structural properties of the brain.

The image preprocessing was performed using statistical parametric mapping (SPM) (Wellcome Department of Cognivite Neurology, http://www.fil.ion.ucl.ac.uk/spm) and FMRIB (Functional Magnetic Resonance Imaging of the Brain) Software Library (FSL) (Smith et al., 2004). Resting state preprocessing consisted of a despiking stage, slice time correction, motion correction, spatial normalization to MNI and smoothing by 6 mm FWHM. The time courses were filtered to the band 0.01–0.1 Hz.

## 3. Methods

We propose a novel Bayesian hierarchical model to predict disease status using imaging data from different modalities, including fALFF, VBM, and FA. For resting-state fMRI scans and DTI scans, the functional and structural information lies in gray matter and white matter, respectively. Most VBM analyses focus on gray matter, which will be the focus of our upcoming data example; however, applications of VBM in white matter has also been found to be associated with psychiatric diseases such as Alzheimer's diseases and schizophrenia (Di et al., 2009; Li et al., 2011). Potentially, our prediction model involves the voxels in gray and/or white matter for different imaging modalities.

### 3.1. Model and estimation

We consider a two-level brain parcellation, initially consisting of *G* = 90 brain regions defined by the automated anatomic labeling (AAL) system (Tzourio-Mazoyer et al., 2002). In each region *g*, we define *L*_*g*_ subregions, ranging from 1 to 9, for *g* = 1, …, *G*. The subregions are built based on the brain parcellation algorithms described in Appendix 1 (Supplementary Material). Each subregion *l* is composed of *V*_*l*_ voxels. Let *X*_*ilg*_(*v*), *Y*_*ilg*_(*v*) and *Z*_*ilg*_(*v*) respectively denote the observed fALFF, FA and VBM measures for subject *i* at voxel *v* in subregion *l* and region *g*, for *i* = 1, …, *n*, *v* = 1, …, *V*_*l*_, *l* = 1, …, *L*_*g*_. Let *N*_*g*_(*l*) ⊆ {1, …, *L*_*g*_} denote the neighboring subregions of subregion *l*, constrained to fall within region *g*, and *n*_*lg*_ is the number of members in *N*_*g*_(*l*). In our model, all the subregions in region *g* are considered as neighbors of subregion *l*; therefore, we have *N*_*g*_(*l*) = {1, …, *L*_*g*_}, and *n*_*lg*_ = *L*_*g*_. Let *D*_*i*_ ∈ {0, 1} denote the disease status (here, PD), where 1 indicates PD; and **W**_*i*_ = (*W*_*i*1_, ⋯, *W*_*iQ*_) denotes the vector of *Q* covariates. Let B, W and G respectively represent the whole brain region, the white matter region and the gray matter region.

We propose a model that accounts for the spatial correlations between voxels within the same subregion, between subregions within the same region, and between regions. Building spatial correlations into our model captures associations between different brain regions and generally improves the precision of estimates by borrowing strength from other (sub)regions. First, our model assumes consistent correlations between voxels in a same subregion. Then the spatial correlations between subregions within the same AAL region are described by a conditional autoregressive (CAR) model, which allows the estimates at subregion levels to borrow strength from their neighbors within the same AAL region. In addition, we introduce unstructured spatial correlations between AAL regions.

Our model reflects the assumption that for each voxel *v* in the gray matter, the fALFF value *X*_*ilg*_(*v*) follows a normal distribution conditioning on the VBM value *Z*_*ilg*_(*v*); for each voxel *v* in the white matter, the FA value *Y*_*ilg*_(*v*) follows a normal distribution conditioning on the VBM value *Z*_*ilg*_(*v*); and for each voxel *v* included in the analysis, the VBM value *Z*_*ilg*_(*v*) follows a normal distribution. The proposed model has the following hierarchical structure:

$$\begin{array}{l} \text{ For any }v\in G,\;[X_{ilg}(v)|Z_{ilg}(v),D_{i},W_{i},•\;]\sim \text{N}\left\{\mu_{lg}^{xz}(v),\delta_{lg}^{xz}\right\},\\ \text{ for any }v\in W,\;[Y_{ilg}(v)|Z_{ilg}(v),D_{i},W_{i},•\;]\sim \text{N}\left\{\mu_{lg}^{yz}(v),\delta_{lg}^{yz}\right\},\\ \text{ for any }v\in ℬ,\;[Z_{ilg}(v)|D_{i},W_{i},•\;]\sim \text{N}\left\{\mu_{lg}^{z}(v),\delta_{lg}^{z}\right\}, \end{array}$$

where

$$\begin{array}{l} \mu_{lg}^{xz}(v)=\sum_{k=0,1}\left[c_{klg}^{xz}(v)(Z_{ilg}(v)-{\bar{Z}}_{lg}(v))+W_{i}\gamma_{klg}^{x}(v)+\beta_{klg}^{x}(v\right)\\ \;+\;\alpha_{ilg}^{x}+\eta_{kg}^{x}]I(D_{i}=k),\\ \mu_{lg}^{yz}(v)=\sum_{k=0,1}\left[c_{klg}^{yz}(v)(Z_{ilg}(v)-{\bar{Z}}_{lg}(v))+W_{i}\gamma_{klg}^{y}(v)+\beta_{klg}^{y}(v\right)\\ \;+\alpha_{ilg}^{y}+\eta_{kg}^{y}]I(D_{i}=k),\\ \mu_{lg}^{z}(v)=\sum_{k=0,1}\left(W_{i}\gamma_{klg}^{z}(v)+\beta_{klg}^{z}(v)+\alpha_{ilg}^{z}+\eta_{kg}^{z})I(D_{i}=k\right). \end{array}$$

We assume that the probability of disease status *P*(*D*_*i*_ = *k*_*i*_) is a constant, and independent of all the parameters. Also, we assume conditional independence among voxel measures of the same modality within the same subregion. The mean structure of the model is composed of several parameters, conditional on disease status. *c*_*klg*_(*v*) is the slope term for centered VBM values; $\gamma_{klg}\left(v\right)={\left(\gamma_{klg1}\left(v\right),\cdots \;,\gamma_{klgQ}\left(v\right)\right)}^{\prime }$ is the parameter vector for covariates; β_*klg*_(*v*), α_*ilg*_, and η_*kg*_ are the voxel-level intercept term, subregion level random effect, and region level intercept term, respectively. Each imaging modality is assumed to have the same subregion-level variance δ_*lg*_ for both subject groups.

The prior beliefs about the parameters included in the likelihood function are expressed in the second or lower levels of the model.

We also assume that

$$\begin{array}{l} c_{klg}^{xz}(v)\sim \text{N}(\zeta_{klg}^{xz},\omega_{klg}^{xz}),\;\zeta_{klg}^{xz}\sim \text{N}(a_{\zeta },b_{\zeta }),\;\omega_{klg}^{xz}\sim \text{InvG}(a_{\omega },b_{\omega }),\\ c_{klg}^{yz}(v)\sim \text{N}(\zeta_{klg}^{yz},\omega_{klg}^{yz}),\;\zeta_{klg}^{yz}\sim \text{N}(a_{\zeta },b_{\zeta }),\;\omega_{klg}^{yz}\sim \text{InvG}(a_{\omega },b_{\omega }),\\ \gamma_{klgq}^{m}(v)\sim \text{N}(0,s_{klg}^{m}),\;s_{klg}^{m}\sim \text{InvG}(a_{s},b_{s}),\\ \beta_{klg}^{m}(v)\sim \text{N}\{\beta_{klg}^{m},\lambda_{klg}^{m}\},\;\lambda_{klg}^{m}\sim \text{InvG}(a_{\lambda },b_{\lambda }), \end{array}$$

The slope *c*_*klg*_(*v*) follows a normal distribution, whose mean and variance are drawn from noninformative hyperpriors. Each covariate parameter γ_*klgq*_(*v*) is assumed to arise from a normal mean-zero distribution with variance *s*_*klg*_, which has a noninformative hyperprior distribution. Parameters β_*klg*_(*v*) that fall within the same subregion are assumed to follow normal distributions with common mean β_*klg*_, and variance λ_*klg*_. We assume a noninformative distribution for λ_*klg*_, and as described in detail below, we use a spatial prior for β_*klg*_ to incorporate spatial correlations between subregions. **η**_*k*_ follows a multivariate normal distribution with mean **0** and covariance matrix **Σ**_*k*_ whose off-diagonal elements capture spatial dependence between AAL regions. Spatial associations between voxels within each subregion are introduced by the individualized random effect term α_*ilg*_, which follows a mean-zero normal distribution with variance τ_*lg*_, thus assuming the same spatial correlations between voxels in the same subregion.

We assume a CAR model for $\beta_{klg}^{m}$ as follows:

$$[\beta_{klg}^{m}|{\left\{\beta_{kl^{\prime }g}^{m}\right\}}_{l^{\prime }\neq l},•\;]\sim \text{N}\left\{\frac{\rho_{g}^{m}}{n_{lg}}\sum_{l^{\prime }\in N_{g}(l)}\beta_{kl^{\prime }g}^{m},\frac{\phi_{g}^{m}}{n_{lg}}\right\},$$

$$\begin{array}{l} \rho_{g}^{m}\sim \text{U}(\{0,0.05,0.1\cdots ,0.8,0.81,\cdots ,0.9,0.91,\cdots ,0.99\}),\\ \;\phi_{g}^{m}\sim \text{InvG}(a_{\phi },b_{\phi }),\\ \alpha_{ilg}^{m}\sim \text{N}(0,\tau_{lg}^{m}),\;\tau_{lg}^{m}\sim \text{InvG}(a_{\tau },b_{\tau }),\\ \eta_{k}^{m}=(\eta_{k1}^{m},\ldots ,\eta_{kG}^{m})\prime \sim \text{N}(0,\Sigma_{k}^{m}),\;\Sigma_{k}^{m}\sim \text{InvW}(\Lambda ,\nu ),\\ \delta_{lg}^{xz}\sim \text{InvG}(a_{\delta },b_{\delta }),\;\delta_{lg}^{yz}\sim \text{InvG}(a_{\delta },b_{\delta }),\\ \delta_{lg}^{z}\sim \text{InvG}(a_{\delta },b_{\delta }),\text{ where }m\in \{x,y,z\}. \end{array}$$

By assuming a subregion level CAR model, we capture the spatial dependence between subregions within each AAL region. In the model, ρ_*g*_ represents the overall degree of spatial dependence in region *g* and $\frac{\phi_{g}}{L_{g}}$ is the conditional variance of β_*klg*_. The neighborhood of subregion *l* ∈ *g*, is defined as all the other subregions in AAL region *g*. The spatial neighborhood effect ρ_*g*_ is assumed to follow a discrete uniform distribution (Gelfand and Vounatsou, 2003). As we would like to identify the similarity of the neighboring subregions, we impose 0 ≤ ρ_*g*_ < 1. Specifically, equal mass is put on the following 36 values: 0, 0.05, 0.1, …, 0.8, 0.81, 0.82, …, 0.90, 0.91, 0.92, …, 0.99, which includes a more refined set of values in the upper range of ρ_*g*_ since estimation of ρ_*g*_ for imaging data often tends toward large values.

For any disease status *k*, the covariance between the voxels within a same subregion *l* in region *g* is contributed by the variance from three components: β_*klg*_, α_*ilg*_, and η_*kg*_; the covariance between the voxels in two subregions *l* and *l*′, but the same AAL region *g*, comes from the covariance between β_*klg*_ and $\beta_{kl^{\prime }g}$, and the variance of η_*kg*_; and the covariance between the voxels in two AAL regions *g* and *g*′ is determined by the covariance of η_*kg*_ and $\eta_{kg^{\prime }}$.

We perform estimation using Markov chain Monte Carlo (MCMC) implemented via Gibbs sampling. The full conditional posterior distributions are shown in Appendix 2 (Supplementary Material).

### 3.2. Prediction

#### 3.2.1. Whole brain prediction

The objective of our model is to predict PD status, given imaging data and other covariates. To achieve this goal, we use the posterior samples drawn from estimation to calculate the posterior predictive probability of disease status.

Let **θ** denote the parameter space, **B**_*i*_ = (**X**_*ilg*_, **Y**_*ilg*_, **Z**_*ilg*_) denote the observed imaging data for subject *i*, and **A**_*i*_ = (**B**_*i*_, *D*_*i*_) denote the combination of the imaging data and disease status. Suppose we have *n* training subjects, and we want to predict the disease status *D*_*n*+1_ for a new subject indexed by *n* + 1. The posterior predictive distribution for *D*_*n*+1_ is given by

(1) $$\begin{array}{l} P(D_{n+1}=k|B_{n+1},{\left\{A_{i}\right\}}_{i=1}^{n})\\ \;=\frac{P(D_{n+1}=k,B_{n+1}|{\left\{A_{i}\right\}}_{i=1}^{n})}{\sum_{k^{\prime }=0,1}P(D_{n+1}=k^{\prime },B_{n+1}|{\left\{A_{i}\right\}}_{i=1}^{n})}\\ \;=\frac{P(D_{n+1}=k)\int_{\theta }P(B_{n+1}|D_{n+1}=k,\theta )P(\theta |{\left\{A_{i}\right\}}_{i=1}^{n})d\theta }{\sum_{k^{\prime }=0,1}P(D_{n+1}=k^{\prime })\int_{\theta }P(B_{n+1}|D_{n+1}=k^{\prime },\theta )P(\theta |{\left\{A_{i}\right\}}_{i=1}^{n})d\theta }, \end{array}$$

where

(2) $$\begin{array}{l} P(B_{n+1}|D_{n+1}=k,\theta )=\prod_{v\in G}P(X_{n+1}(v)|Z_{n+1}(v),D_{n+1}=k,\theta )\\ \;P(Z_{n+1}(v)|D_{n+1}=k,\theta )\\ \;\prod_{v\in W}P(Y_{n+1}(v)|Z_{n+1}(v),D_{n+1}=k,\theta )\\ \;P(Z_{n+1}(v)|D_{n+1}=k,\theta ), \end{array}$$

Suppose we draw *T* posterior samples, denoted **θ**^(*t*)^, from $P\left(\theta |{\left\{{\text{A}}_{i}\right\}}_{i=1}^{n}\right)$, for *t* = 1, ⋯, *T*. Letting $\pi_{k}^{\left(t\right)}=P\left({\text{B}}_{n+1}|D_{n+1}=k,\theta^{\left(t\right)}\right)$, the posterior predictive probability can be expressed by

(3) $$\hat{P}(D_{n+1}=k|B_{n+1},{\left\{A_{i}\right\}}_{i=1}^{n})=\frac{P(D_{n+1}=k)\sum_{t=1}^{T}\pi_{k}^{\left(t\right)}}{\sum_{k^{\prime }=0,1}P(D_{n+1}=k^{\prime })\sum_{t=1}^{T}\pi_{k}^{\left(t\right)}}.$$

Then ultimately the prediction of *D*_*n*+1_ is given by

(4) $${\hat{D}}_{n+1}=\arg\;\underset{k}{\max}\left(P(D_{n+1}=k)\sum_{t=1}^{T}\pi_{k}^{\left(t\right)}\right).$$

To evaluate the performance of our method, we calculate the prediction accuracy using LOOCV.

Applied directly, LOOCV is very computational expensive because it involves multiple posterior simulations with tens of thousands voxels included in the analysis. Therefore, we employ an importance sampling approach to reduce the computation for LOOCV of our model (Gelfand et al., 1992; Gelfand, 1996; Alqallaf and Gustafson, 2001; Vehtari and Lampinen, 2002). Specifically, the LOOCV predictive probabilities can be expressed by

(5) $$P(D_{i}=k|B_{i},A_{-i})=\frac{P(D_{i}=k)Q_{kd_{i}}}{\sum_{k^{\prime }=0,1}P(D_{i}=k^{\prime })Q_{k^{\prime }d_{i}}},$$

where

(6) $$Q_{kd_{i}}=\int \frac{P(B_{i}|D_{i}=k,\theta )}{P(B_{i}|D_{i}=d_{i},\theta )}P(\theta |{\left\{A_{i}\right\}}_{i=1}^{n})d\theta ,$$

and *d*_*i*_ is the observed disease status for subject *i*. Next, we provide the details of how *Q*_*kd*_*i*__ is derived. The posterior predictive probability can be written as follows:

(7) $$\begin{array}{l} P(D_{i}=k|B_{i},A_{-i})\\ \;=\int P(D_{i}=k|B_{i},\theta )\frac{P(\theta |B_{i},A_{-i})}{P(\theta |B_{i},D_{i}=d_{i},A_{-i})}\\ \;P(\theta |B_{i},D_{i}=d_{i},A_{-i})d\theta . \end{array}$$

Therefore,

(8) $$\frac{P(D_{i}=k|B_{i},A_{-i})}{P(D_{i}=d_{i}|B_{i},A_{-i})}:=\frac{P(D_{i}=k)}{P(D_{i}=d_{i})}Q_{kd_{i}}.$$

By using the fact that $\underset{k=0,1}{\sum }P\left(D_{i}=k|{\text{B}}_{i},{\text{A}}_{-i}\right)=1$, we have

(9) $$P(D_{i}=d_{i}|B_{i},A_{-i})=\frac{P(D_{i}=d_{i})}{\sum_{k=0,1}P(D_{i}=k)Q_{kd_{i}}},$$

thus leading to the above LOOCV predictive probability Equation (5). For *i* = 1, ⋯ , *n* and *k* = 0, 1, compute

(10) $${\hat{Q}}_{kd_{i}}=\frac{1}{T}\sum_{t=1}^{T}\frac{P(B_{i}|D_{i}=k,\theta^{\left(t\right)})}{P(B_{i}|D_{i}=d_{i},\theta^{\left(t\right)})}.$$

The estimate of *D*_*i*_ is

(11) $${\hat{D}}_{i}=\arg\;\underset{k}{\max}\left(P(D_{i}=k)Q_{kd_{i}}\right).$$

Since there are only two possible values for *D*_*i*_, we only need to calculate $P\left({\text{B}}_{i}|D_{i}=k,\theta^{\left(t\right)}\right)$ and $P\left({\text{B}}_{i}|D_{i}=d_{i},\theta^{\left(t\right)}\right)$, where *k*≠*d*_*i*_, for each subject *i*.

#### 3.2.2. Voxel-level prediction

We also consider the use of imaging data **B**_*i*_(*v*) = (*X*_*ilg*_(*v*), *Y*_*ilg*_(*v*), *Z*_*ilg*_(*v*)) for subject *i* at voxel *v* to predict the disease status *D*_*i*_. Similar to Equation (5), the voxel-level LOOCV predictive probabilities can be expressed by

(12) $$P(D_{i}=k|B_{i}(v),A_{-i})=\frac{P(D_{i}=k)Q_{kd_{i}}}{\sum_{k^{\prime }=0,1}P(D_{i}=k^{\prime })Q_{k^{\prime }d_{i}}},$$

where

(13) $$Q_{kd_{i}}=\int \frac{P(B_{i}(v)|D_{i}=k,\theta )/\sum_{k^{\prime }=0,1}P(B_{i}(v)|D_{i}=k^{\prime },\theta )P(D_{i}=k^{\prime })}{P(B_{i}|D_{i}=d_{i},\theta )/\sum_{k^{\prime }=0,1}P(B_{i}|D_{i}=k^{\prime },\theta )P(D_{i}=k^{\prime })}P(\theta |{\left\{A_{i}\right\}}_{i=1}^{n})d\theta ,$$

which is estimated by

(14) $${\hat{Q}}_{kd_{i}}=\frac{1}{T}\sum_{t=1}^{T}\frac{P(B_{i}(v)|D_{i}=k,\theta^{\left(t\right)})/\sum_{k^{\prime }=0,1}P(B_{i}(v)|D_{i}=k^{\prime },\theta^{\left(t\right)})P(D_{i}=k^{\prime })}{P(B_{i}|D_{i}=d_{i},\theta^{\left(t\right)})/\sum_{k^{\prime }=0,1}P(B_{i}|D_{i}=k^{\prime },\theta^{\left(t\right)})P(D_{i}=k^{\prime })}.$$

Then the estimate of *D*_*i*_ is

(15) $${\hat{D}}_{i}=\arg\;\underset{k}{\max}\left(P(D_{i}=k)Q_{kd_{i}}\right),$$

which is equivalent to

(16) $${\hat{D}}_{i}=\arg\;\underset{k}{\max}\left(P(D_{i}=k)\frac{1}{T}\sum_{t=1}^{T}P(B_{i}(v)|D_{i}=k,\theta^{\left(t\right)})\right).$$

*Q*_*kd*_*i*__ is derived in the similar way as in the whole brain analysis. The derivation of Equation (14) is described in Appendix 3 (Supplementary Material).

The voxel-level prediction result can be used as a way to select the regions that are highly associated with PD if the prediction accuracy is high in these regions. An alternative approach to perform feature selection using our model is discussed in section 5.

## 4. Results

### 4.1. Parkinson's disease data

We applied our proposed Bayesian spatial model to PD data, which has T1 and resting-state fMRI images available; therefore, our model reduces to one which includes two imaging modalities, VBM and fALFF, and only considers data in the gray matter. We generate predictions of PD based on multimodal imaging data aggregated across the whole brain, and we provide voxel-level predictions as well. By evaluating the prediction accuracy at each voxel, we are able to identify brain regions that are highly associated with Parkinson's disease as an alternative to performing feature selection.

In the estimation procedure, the hyperparameters for the prior distribution are set to provide vague information to ensure that the results are dominated by the information in the data. Specifically, all the hyperparameters in the inverse-gamma distribution are set to 10^−3^ (Spiegelhalter et al., 1994/2003), the normal prior for ζ_*klg*_ is assumed to have mean *a*_ζ_ = 0 and variance $b_{\zeta }=10^{5}$. In the inverse-Wishart distribution, the degrees of freedom ν should be greater than *G*−1 to build a proper distribution, so we set ν = *G*, which provides the least information based on our data. The scale matrix Λ is set as $10^{-3}\times {\text{I}}_{G}$, where **I**_*G*_ is a *G* × *G* identity matrix.

We perform a total of 10,000 MCMC iterations including 5,000 burn-in iterations, and store the results thinning by 10. Due to the massive number of parameters in our model, we randomly check trace plots for parameters at the voxel-level, subregion-level, and region-level, respectively. We provide some examples in Appendix 4 (Supplementary Material).

After estimating the model parameters, we perform a whole-brain and voxel-level prediction using posterior samples based on procedures described in section 3.2. Here, we have a total of 500 posterior samples after thinning. By assuming an equal probability for classification as a PD patient and a control subject, our model achieves 100% accuracy from the whole-brain prediction based on LOOCV.

**Figure 1:**
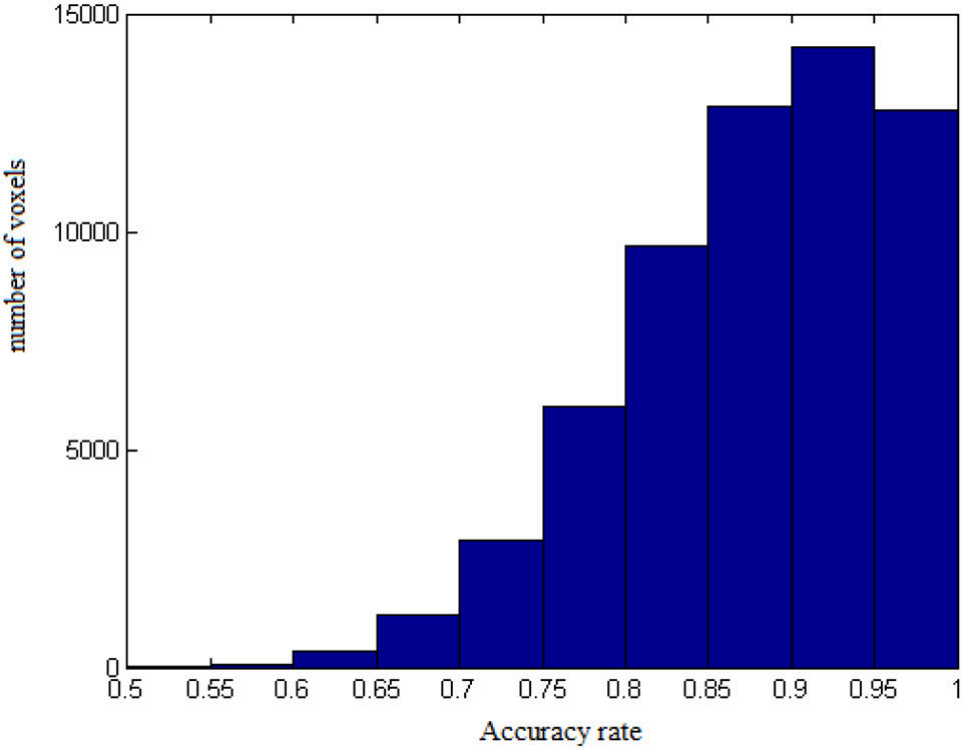
The distribution of average accuracy rates for prediction across subjects for all the voxels included in the analyses.

**Figure 2:**
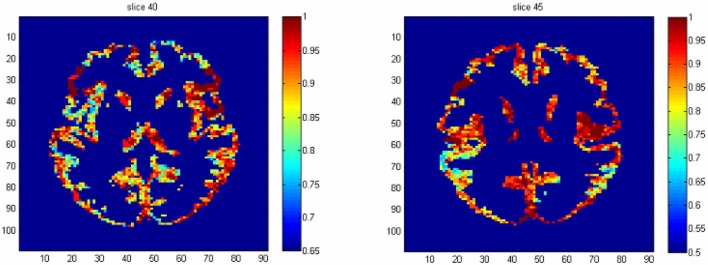
The average prediction map based on the voxel-level prediction results across subjects.

The results from voxel-level prediction provide interesting information as well. The highest voxel-level accuracy rate is 100%, and the lowest is near 50%. Figure 1 shows the distribution of the average accuracy rate across subjects for all the voxels included in the analysis. Table 1 gives the number of voxels (percentage) achieving accuracy rates higher than 80%. Also, an average whole-brain prediction map based on the results from voxel-level prediction across subjects are presented in Figure 2.

**Table 1:** Summary of average accuracy rates for prediction across subjects.

**Accuracy rate**	**[80%, 85%)**	**[85%, 90%)**	**[90%, 95%)**	**[95%, 100%)**	**100%**
Number of voxels	5,993	9,663	12,878	14,236	12,764
(Percentage)	(9.97)	(16.07)	(21.42)	(23.68)	(21.23)

**Figure 3:**
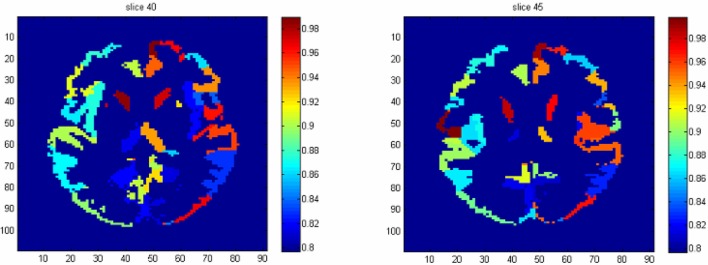
The region-level prediction map based on the average accuracy rates across voxels within a region.

To identify the regions which are predictive for disease status, we compute the average accuracy rates across voxels within a region, and Table 2 shows the regions that have accuracy rate above 95%. Table 2 also shows the percentage of voxels exceeding 90% accuracy rates for those regions. The right rectus gyrus, which is associated with cognitive impairment in PD patients, and is shown to have different gray matter density between PD and controls (Nagano-Saito et al., 2005), is identified in our analysis. The precentral gyrus, which is part of the primary motor cortex, is identified among the most accurate brain regions, and its performance is consistent with the involvement of this region in planning and initiating motor movements, which is critically impaired in patients with PD. We also find the bilateral caudate and the left putamen as regions with accurate predictions. The caudate and putamen, two regions comprising the dorsal striatum, exhibit marked pathologic changes from PD, linked to the loss of dopaminergic neurons in the substantia nigra which projects to striatal neurons in the caudate nucleus and putamen (Spencer et al., 1992). The right fusiform gyrus, which is believed to related to impaired ability to correctly identify negative facial expressions (Geday et al., 2006), and the left inferior parietal lobule which is involved in the perception of emotions in facial stimuli, may play a role of differentiating healthy controls and PD patients as well. Other regions which are involved in face perception such as the right mid-temporal pole are also identified. The left postcentral gyrus, the left superior parietal lobule, and the right superior medial frontal gyrus also stand out since all of them are part of the sensory system. A region-level prediction map based on the average accuracy rates across voxels within a region is shown in Figure 3.

**Table 2:** List of regions with above 95% average accuracy rate across voxels.

**Region**	**Accuracy rate**	**Percentage of voxels with accuracy rate > 90%**
Left postcentral	99.9%	42.6%
Right rectus	99.3%	52.2%
Left inferior parietal	99.3%	90.2%
Right superior medial frontal	99.0%	61.1%

### 4.2. Simulation studies

We conduct a simulation study to evaluate the performance of our proposed model. The purpose of this simulation study is to show that the MCMC generated samples from our model accurately target the true values and that the whole-brain prediction is accurate. In addition, we demonstrate that our model can distinguish regions that are predictive of disease status.

We assume that the imaging data are generated from the likelihood function of our model. We simulate data for 25 subjects from three AAL regions, the number of subregions within an AAL region has a mean and variance of 3, and the number of voxels within a subregion has a mean and variance of 50. We specify the true values for the parameters in the likelihood function, i.e., *c*_*klg*_(*v*), γ_*klg*_(*v*), β_*klg*_(*v*), α_*ilg*_, η_*kg*_, and δ_*lg*_, which are the most relevant parameters for voxel-level inference and future prediction. In this way, we can compare our posterior estimations with specified true values. All the other parameters are updated from the posterior distributions. And the hyperparameters are set to be the same as in data from PD study. We select some subregions to be the ones that are associated with PD, and a region is classified into this category if it contains those selected subregions. We set different true values of parameters for disease and non-disease group if they are within the pre-specified regions and otherwise assume that the true values are the same the for two groups. A total of 100 data sets are drawn in the simulation study. The programming is implemented in Matlab, and the computation is performed on a Linux cluster with 16 GB of RAM. Execution time is approximately 3–4 h for one data set.

First, we evaluate the posterior estimates by comparing the posterior means to the true values. Instead of examining a total of five thousand parameters which have known true values separately, we calculate the mean structure and variance of the likelihood function from posterior samples and compare them to the truth since they are the most essential for inferences and predictions. The average bias (percentage change) in mean structure is 3.52 × 10^−2^ (0.54%), and in variance is 1.04 × 10^−5^ (1.04%). Secondly, we calculate the accuracy of whole-brain prediction. The LOOCV achieves 100% for the whole-brain prediction for all 100 simulated data sets. Thirdly, we identify the regions that are highly associated with disease status by evaluating the voxel-level accuracy rates for prediction. We compare the average accuracy for voxel-level prediction between the pre-specified regions and the others. Within the pre-specified regions, the average accuracy rate is 99.8%; for voxels which are in the other regions, the average accuracy rate is 71.7%. Here, we can see an improvement in prediction when voxels are from the pre-specified regions.

In comparison, we apply the elastic-net model to the simulated data as described above, and the LOOCV achieves an average of 86% accuracy rate for the whole-brain prediction.

In summary, our model accurately performs posterior estimation with small bias, provides accurate prediction of PD status using whole-brain imaging data, and correctly identifies the regions that are highly associated with disease.

## 5. Discussion

We propose a Bayesian spatial model to predict PD using different modalities of imaging data, including fALFF, VBM, and FA in gray and white matter. Our framework performs voxel-level estimation for imaging data and conducts whole-brain and voxel-level prediction of disease status based on posterior predictive probabilities. Our model estimates both the mean and covariance structures of imaging data, predicting disease status using whole-brain imaging data, and identifying the regions which are highly associated with the disease based on voxel-level prediction results.

In our framework, we consider spatial correlations at voxel level, subregion level, and region level, and specify different correlation structures such as exchangeable, CAR, and unstructured correlation matrices for them. The rich hierarchical spatial correlation structures captured by our model extends previous spatial modeling frameworks by Bowman et al. (2008) and Derado et al. (2013). The intra-subregion correlation in our model is described by a single value within each subregion; the inter-subregion correlation is modeled by a CAR model which borrows information from the subregions within the same parent AAL region; the inter-region correlation is assumed to have a unstructured correlation matrix.

We derive the posterior predictive probability using whole brain data and data from a single voxel. Due to the complexity of computation, we adopt an importance sampling strategy to conduct LOOCV. The importance sampling techniques estimate the LOOCV error rate based only on one-model fitting using all samples and produces very accurate estimate on the LOOCV error rate. We evaluate the accuracy rate of the whole-brain prediction and identify the regions that are predictive for disease based on the results from voxel-level accuracy rates. Our model accounts for spatial correlations embedded in the data; however, additional multiple testing strategies could be explored to account for potential dependence inherent in the data. Our model increases localization compared to some approaches by offering voxel-level predictions. While we incorporate information from multiple modalities, we are unable to dissociate the relative predictive accuracy generated by each modality.

One weakness of our method is computational time since we use a joint model that performs estimation at the voxel-level. However, by applying the importance sampling strategy, we only need to perform the posterior estimation once, and then the posterior predictive probabilities can be computed fairly efficiently.

Compared to the existing feature selection methods, e.g., LASSO or elastic-net, our model uses a different modeling strategy and different criteria for selection. LASSO and elastic-net model the probability of PD, while our method starts from modeling the imaging data. This distinction leads to an important advantage that we are able to estimate and borrow strength from the spatial correlations in the data, whereas highly correlated predictors often lead to poor performance of the LASSO and related methods. Also, we use posterior predictive probability as the criteria to select the features, which is the exact target of prediction problems; on the other hand, LASSO and elastic-net, from a Bayesian perspective, use posterior modes to perform feature selection. Our model also has interpretive advantages over SVM and GP models by identifying particular voxels, subregions, or regions that contribute significantly to accurate prediction. Compared to the methodology of scalar-on-image regression (Goldsmith et al., 2014; Reiss et al., 2015; Kang et al., 2016; Wang et al., 2017), our method models the images as the response, which is a natural generative process, and then we predict the disease distribution given the imaging scans.

In summary, the advantages of our proposed Bayesian method are three fold. First, it is more straightforward to incorporate prior knowledge regarding brain function and structure, which is extremely useful to improve the prediction accuracy and to provide a better understanding of the etiology. Second, it yields estimates and inference from the full posterior distribution, e.g., rather than point estimates. In particular, it can provide measures of uncertainty of the predictions based on the posterior predictive distribution. In addition, the posterior computation based on the MCMC algorithm is more robust to complex imaging data, while the optimization algorithms for other frequentist prediction methods are more likely to be trapped at the local modes, which may reduce the prediction accuracy.

In our method, we select features based on the posterior predictive probability of each voxel; ideally, we would like to identify the voxels v∈V s.t.

(17) $$P(D_{i}=k|{\left\{B_{i}(v)\right\}}_{v\in V},A_{-i})=P(D_{i}=k|B_{i},A_{-i}),$$

which could be a possible extension of our proposed approach.

For Parkinson's disease, our model may not immediately supplant current clinical standards to diagnose patients at or near the manifestation of motor symptoms. However, our model stands to provide insights into the useful information for the diagnosis of PD, underlying neurophysiological basis of the disease, potentially early pre-motor alterations, and effective strategies to design studies examining potential neuroprotective treatments with consideration of the cost and complexity as well as extensive validation and comparison to current standards.

## Author contributions

WX: methodology, simulation, and writing. FB and JK: supervising and editing the manuscript.

### Conflict of interest statement

The authors declare that the research was conducted in the absence of any commercial or financial relationships that could be construed as a potential conflict of interest.
