# Supplementary material for: A Bayesian Spatial Model to Predict Disease Status Using Imaging Data From Various Modalities
Source: Front Neurosci. 2018 Mar 26;12:184. doi: 10.3389/fnins.2018.00184 (PMC5879954; doi:10.3389/fnins.2018.00184)

Appendix

1. Brain parcellation We consider a brain parcellation that is a more granular version of the AAL regional template; we utilize the existing boundaries but provide a further subdivision within each region of interest. A hierarchical clustering algorithm is applied to each region, where the spatial contiguity of the resulting clusters is enforced using the distance matrix. Connectivity information, averaged across the sample, is utilized to subtly promote functional and structural homogeneity in the grey and white matter, respectively, of each resulting cluster. Finally, we incorporate tissue-type information (grey/white matter) in the distance matrix to encourage balance in the tissue composition of clusters. Based on average linkage, the number of clusters is chosen manually to yield a resulting parcellation consisting of approximately 200 regions (here, $\sum_{g=1}^{G} L_g = 282$ regions are selected), using the Hierarchical Clustering tools in MATLAB and Statistics Toolbox Release 2012b (The MathWorks, Inc., Natick, Massachusetts, United States). Independently for each AAL region, let d_{ij} represent the Manhattan distance, which is the sum of the absolute differences of MNI coordinates, for voxels i and j, and define the distance matrix as:

$$D_{ij} = \begin{cases} d_{ij} + (1 - \mathrm{FC}_{ij}) & \text{if } i \in \mathcal{G}, j \in \mathcal{G} \\ d_{ij} + (1 - \mathrm{SC}_{ij}) & \text{if } i \in \mathcal{W}, j \in \mathcal{W} \\ d_{ij} & \text{otherwise,} \end{cases}$$

where FC_{ij} represents the average of functional connectivity for voxel pairs in grey matter $\mathcal{G}$ and SC_{ij} represents the average of structural connectivity for voxel pairs in white matter $\mathcal{W}$. Functional connectivity is quantified by estimating the Pearson correlation coefficient between the fMRI-derived BOLD signals of grey The prior beliefs about the parameters included in the likelihood function are expressed in the second or lower levels of the model. matter voxel pairs for each subject, and averaging across subjects. Structural connectivity is quantified by calculating the correlation between "connectivity profiles" and subsequently averaging across subjects. The "connectivity profile" for a white matter voxel is obtained by using a set of predefined target regions scattered throughout the brain and applying probabilistic tractography tools in FSL (Behrens, 2003) to each subject's DTI data, yielding values that reflect the strength of structural connectivity between the voxel and each of the target regions. Thus, SC_{ij} is expected to reflect the average consistency in structural connections between white matter voxel pairs. For tissue-heterogenous voxel pairs, there is no obvious way to quantify connectivity; by using an unpenalized distance of d_{ij} for these mismatched voxel pairs, the resulting clusters tend to span both grey and white matter.

2. Posterior distributions

We present the full conditional posterior distributions for Gibbs sampling. Here, we take the parameters with superscript xz as examples, parameters with other superscripts can be derived similarly. Let $\overline{\xi}$ denote all the parameters except ξ. Denote $\tilde{Z}_{ilg}(v) = Z_{ilg}(v) - \bar{Z}_{lg}(v)$. Then the full conditional posterior distributions can be derived as follows:

(1) $[c_{klg}(v) \mid \mathbf{X}_{lg}(v), \mathbf{Y}_{lg}(v), \mathbf{Z}_{lg}(v), \mathbf{D}, \mathbf{W}, \overline{c}_{klg}(v)\ ]$

$$\propto \mathrm{N}\left(\mu_{c_{klg}(v)}, \sigma^2_{c_{klg}(v)}\right)$$

$$\mu_{c_{klg}(v)} = \frac{\omega_{klg}\sum_{i=1}^{n} I(D_i = k)\tilde{Z}_{ilg}(v)\left\{X_{ilg}(v) - \mathbf{W}_i\boldsymbol{\gamma}_{klg} - \beta_{klg}(v) - \alpha_{ilg} - \eta_{kg}\right\} + \delta_{lg}\zeta_{klg}}{\omega_{klg}\sum_{i=1}^{n} I(D_i = k)\tilde{Z}_{ilg}(v)^2 + \delta_{lg}}$$

$$\sigma^2_{c_{klg}(v)} = \frac{\delta_{lg}\omega_{klg}}{\omega_{klg}\sum_{i=1}^{n} I(D_i = k)\tilde{Z}_{ilg}(v)^2 + \delta_{lg}}$$

(2) $[\gamma_{klgq}(v) \mid \mathbf{X}_{lg}(v), \mathbf{Y}_{lg}(v), \mathbf{Z}_{lg}(v), \mathbf{D}, \mathbf{W}, \overline{\gamma}_{klgq}(v)\ ]$

$$\propto \mathrm{N}\left(\mu_{\gamma_{lgq}(v)}, \sigma^2_{\gamma_{lgq}(v)}\right)$$

$$\mu_{\gamma_{lgq}(v)} = \frac{s_{klg}\displaystyle\sum_{i=1}^{n} I(D_i = k)\left(W_{iq}[X_{ilg}(v) - c_{klg}(v)\tilde{Z}_{ilg}(v) - \beta_{klg}(v) - \alpha_{ilg} - \eta_{kg} - \sum_{q'\neq q} W_{iq'}\gamma_{klgq'}(v)]\right)}{s_{klg}\sum_{i=1}^{n} I(D_i = k)W_{iq}^2 + \delta_{lg}}$$

$$\sigma^2_{\gamma_{lgq}(v)} = \frac{\delta_{lg}s_{klg}}{s_{klg}\sum_{i=1}^{n} I(D_i = k)W_{iq}^2 + \delta_{lg}}$$

(3) $[\beta_{klg}(v) \mid \mathbf{X}_{lg}(v), \mathbf{Y}_{lg}(v), \mathbf{Z}_{lg}(v), \mathbf{D}, \mathbf{W}, \overline{\beta}_{klg}(v)\ ]$

$$\propto \mathrm{N}\left(\mu_{\beta_{klg}(v)}, \sigma^2_{klg}(v)\right)$$

$$\mu_{\beta_{klq}(v)} = \frac{\lambda_{klg}\sum_{i=1}^{n} I(D_i = k)[X_{ilg}(v) - c_{klg}(v)\tilde{Z}_{ilg}(v) - \mathbf{W}_i\boldsymbol{\gamma}_{klg} - \alpha_{ilg} - \eta_{kg}] + \beta_{klg}\delta_{lg}}{\lambda_{klg}\sum_{i=1}^{n} I(D_i = k) + \delta_{lq}}$$

$$\sigma^2_{\beta_{klg}(v)} = \frac{\delta_{lg}\lambda_{klg}}{\lambda_{klg}\sum_{i=1}^{n} I(D_i = k) + \delta_{lg}}$$

(4) $[\alpha_{ilg} \mid X_{ilg}(v), Y_{ilg}(v), Z_{ilg}(v), D_i, \mathbf{W}_i, \overline{\alpha}_{ilg}\ ]$

$$\propto \mathrm{N}\left\{\frac{\tau_{lg}\sum_{v\in l}[X_{ilg}(v) - c_{klg}(v)\tilde{Z}_{ilg}(v) - \mathbf{W}_i\boldsymbol{\gamma}_{klg} - \beta_{klg}(v) - \eta_{kg}]}{\tau_{lg}V_{lg} + \delta_{lg}}, \frac{\delta_{lg}\tau_{lg}}{\tau_{lg}V_{lg} + \delta_{lg}}\right\}$$

(5) $[\delta_{lg} \mid \mathbf{X}_{lg}(v), \mathbf{Y}_{lg}(v), \mathbf{Z}_{lg}(v), \mathbf{D}, \mathbf{W}, \overline{\delta}_{lg}\ ]$

$$\propto \mathrm{InvG}\,(a, b)$$

$$a = a_\delta + \frac{nV_{lg}}{2}$$

$$b = b_\delta + \frac{\displaystyle\sum_{i=1}^{n}\sum_{v\in l}\left\{X_{ilg}(v) - [c_{klg}(v)\tilde{Z}_{ilg}(v) + \mathbf{W}_i\boldsymbol{\gamma}_{klg} + \beta_{klg}(v) + \alpha_{ilg} + \eta_{kg}]\right\}^2}{2}$$

$$(6)\ [\zeta_{klg} \mid \mathbf{X}_{lg}(v), \mathbf{Y}_{lg}(v), \mathbf{Z}_{lg}(v), \mathbf{D}, \mathbf{W}, \overline{\zeta}_{klg}\ ]$$
$$\propto \mathrm{N}\left(\frac{b_\zeta \sum_{v\in l} c_{klg}(v) + \omega_{klg} a_\zeta}{V_{lg} b_\zeta + \omega_{klg}}, \frac{\omega_{klg} b_\zeta}{V_{lg} b_\zeta + \omega_{klg}}\right)$$

$$(7)\ [\omega_{klg} \mid \mathbf{X}_{lg}(v), \mathbf{Y}_{lg}(v), \mathbf{Z}_{lg}(v), \mathbf{D}, \mathbf{W}, \overline{\omega}_{klg}\ ]$$
$$\propto \mathrm{InvG}\left(a_\omega + \frac{V_{lg}}{2}, b_\omega + \frac{\sum_{v\in l} \left(c_{klg}(v) - \zeta_{klg}\right)^2}{2}\right)$$

$$(8)\ [s_{klg} \mid \mathbf{X}_{lg}(v), \mathbf{Y}_{lg}(v), \mathbf{Z}_{lg}(v), \mathbf{D}, \mathbf{W}, \overline{s}_{klg}\ ]$$
$$\propto \mathrm{InvG}\left(a_s + \frac{Q \times V_{lg}}{2}, b_s + \frac{\sum_{v\in l} \sum_{q=1}^{Q} \gamma_{klgq}^2(v)}{2}\right)$$

$$(9)\ [\beta_{klg} \mid \mathbf{X}_{lg}(v), \mathbf{Y}_{lg}(v), \mathbf{Z}_{lg}(v), \mathbf{D}, \mathbf{W}, \overline{\beta}_{klg}\ ]$$
$$\propto \mathrm{N}\left(\frac{\phi_g \sum_{v\in l} \beta_{klg}(v) + \lambda_{klg} \rho_g \sum_{l' \in N_g(l)} \beta_{kl'g}}{V_{lg}\phi_g + n_{lg}\lambda_{klg}}, \frac{\lambda_{klg}\phi_g}{V_{lg}\phi_g + n_{lg}\lambda_{klg}}\right)$$

$$(10)\ [\lambda_{klg} \mid \mathbf{X}_{lg}(v), \mathbf{Y}_{lg}(v), \mathbf{Z}_{lg}(v), \mathbf{D}, \mathbf{W}, \overline{\lambda}_{klg}\ ]$$
$$\propto \mathrm{InvG}\left(a_\lambda + \frac{V_{lg}}{2}, b_\lambda + \frac{\sum_{v\in l} \left(\beta_{klg}(v) - \beta_{klg}\right)^2}{2}\right)$$

$$(11)\ [\tau_{lg} \mid \mathbf{X}_{lg}(v), \mathbf{Y}_{lg}(v), \mathbf{Z}_{lg}(v), \mathbf{D}, \mathbf{W}, \overline{\tau}_{lg}\ ]$$
$$\propto \mathrm{InvG}\left(a_\tau + \frac{n}{2}, b_\tau + \frac{\sum_{i=1}^{n} \alpha_{ilg}^2}{2}\right)$$

(12) $[\phi_g \mid \mathbf{X}_{lg}(v), \mathbf{Y}_{lg}(v), \mathbf{Z}_{lg}(v), \mathbf{D}, \mathbf{W}, \overline{\phi}_g\ ]$

$$\propto \text{InvG}\left(a_\phi + L_g, b_\phi + \frac{\sum_{k=0,1}\sum_{l\in g} n_{lg}\left(\beta_{klg} - \frac{\rho_g}{n_{lg}}\sum_{l'\in N_g(l)}\beta_{kl'g}\right)^2}{2}\right)$$

(13) $[\rho_g \mid \mathbf{X}_{lg}(v), \mathbf{Y}_{lg}(v), \mathbf{Z}_{lg}(v), \mathbf{D}, \mathbf{W}, \overline{\rho}_g\ ]$

$$\propto \exp\left\{-\frac{1}{2\phi_g}\left(\rho_g^2 \sum_{k=0,1}\sum_{l\in g}\frac{\left(\sum_{l'\in N_g(l)}\beta_{kl'g}\right)^2}{n_{lg}} - 2\rho_g \sum_{k=0,1}\sum_{l\in g}\beta_{klg}\sum_{l'\in N_g(l)}\beta_{kl'g}\right)\right\}$$
$$\propto L(\rho_g)$$

Suppose ρ_g follows a discrete uniform distribution. In our analysis, ρ_g can take 36 values $\{m_1, \cdots m_n\}$. Denote the posterior probability of ρ_g as $\{p_1, \cdots, p_n\}$, then

$$p_j = \frac{L(\rho_g = m_j)}{L(\rho_g = m_1) + \cdots + L(\rho_g = m_n)}.$$

(14) Let $\boldsymbol{\beta}_{kg} = \left(\beta_{k1g}(v_1), \cdots, \beta_{k1g}(v_{V_{1g}}), \beta_{k2g}(v_1)\cdots, \beta_{kL_gg}(v_{V_{L_g}g})\right)^T$ denote all the voxels in region g; define $\mathbf{c}_{kg}$, $\boldsymbol{\gamma}_{kg}$, $\mathbf{X}_{ig}$ and $\mathbf{Z}_{ig}$ in the same way; $V_g = \sum_{l=1}^{L_g} V_{lg}$ denote the number of voxels in region g; $\boldsymbol{\alpha}_{ig} = (\alpha_{i1g}, \cdots, \alpha_{iL_gg})^T$; $\boldsymbol{\Delta}_g = diag(\boldsymbol{\Delta}_{1g}, \cdots, \boldsymbol{\Delta}_{L_gg})$, where $\boldsymbol{\Delta}_{lg} = \mathbf{I}_{V_{lg}} \otimes \delta_{lg}$; $\odot$ denote Hadamard product.

$$[\boldsymbol{\eta}_k \mid \mathbf{X}_{lg}(v), \mathbf{Y}_{lg}(v), \mathbf{Z}_{lg}(v), \mathbf{D}, \mathbf{W}, \overline{\boldsymbol{\eta}}_k\ ]$$
$$\propto \exp\left\{-\frac{1}{2}\boldsymbol{\eta}_k^T\boldsymbol{\Sigma}_k^{-1}\boldsymbol{\eta}_k\right\}$$
$$\exp\left\{-\frac{1}{2}\sum_{i=1}^{N}\sum_{g=1}^{G}\ \left\{\sum_{k=0,1}\mathbf{1}_{V_g}\eta_{kg}I(D_i = k) - \right.\right.$$
$$\left.\underbrace{\left(\mathbf{X}_{ig} - \sum_{k=0,1}\left\{\mathbf{c}_{kg}\odot\tilde{\mathbf{Z}}_{ig} + \mathbf{W}_i\gamma_{kg} + \boldsymbol{\beta}_{kg} + \left(\mathbf{1}_{V_{1g}}^T, \cdots, \mathbf{1}_{V_{L_g g}}^T\right)^T \otimes \boldsymbol{\alpha}_{ig}\right\} I(D_i = k)\right)}_{=:\mathbf{t}_g}\right\}^T$$
$$\left.\boldsymbol{\Delta}_g^{-1}\left(\sum_{k=0,1}\mathbf{1}_{V_g}\eta_{kg}I(D_i = k) - \mathbf{t}_g\right)\right\}$$

$$
\begin{aligned}
\propto & \exp\left\{-\frac{1}{2}\boldsymbol{\eta}_k^T\boldsymbol{\Sigma}_k^{-1}\boldsymbol{\eta}_k\right\} \\
& \exp\left\{-\frac{1}{2}\underbrace{\sum_{i=1}^{N}\sum_{g=1}^{G}\left\{\sum_{k=0,1}\mathbf{1}_{V_g}\eta_{kg}I(D_i=k)\right\}^T\boldsymbol{\Delta}_g^{-1}\left\{\sum_{k=0,1}\mathbf{1}_{V_g}\eta_{kg}I(D_i=k)\right\}}_{I}\right. \\
& \left.-\underbrace{\sum_{i=1}^{N}\sum_{g=1}^{G}\left\{\sum_{k=0,1}\mathbf{1}_{V_g}\eta_{kg}I(D_i=k)\right\}^T\boldsymbol{\Delta}_g^{-1}\mathbf{t}_g}_{II}\right\}
\end{aligned}
$$

$$
\begin{aligned}
I =& \boldsymbol{\eta}_k^T diag\left\{\underbrace{\sum_{i=1}^{n}I(D_i=k)\sum_{l=1}^{L_1}[\delta_{l1}^{-1}V_{l1}],\cdots,\sum_{i=1}^{n}I(D_i=k)\sum_{l=1}^{L_G}[\delta_{l1}^{-1}V_{lG}]}_{\boldsymbol{\Omega}_{\boldsymbol{\eta}_k}}\right\}\boldsymbol{\eta}_k \\
II =& \boldsymbol{\eta}_k^T\underbrace{\left(P_{\boldsymbol{\eta}_{k1}},\cdots,P_{\boldsymbol{\eta}_{kG}}\right)'}_{\mathbf{P}_{\boldsymbol{\eta}_k}} \\
P_{\boldsymbol{\eta}_{kg}} =& \sum_{i=1}^{n}I(D_i=k)\sum_{l=1}^{L_g}[\delta_{lg}^{-1}\sum_{v=1}^{V_{lg}}\left(X_{ilg}(v)-c_{klg}(v)\tilde{Z}_{ilg}(v)-\mathbf{W}_i\gamma_{klg}-\beta_{klg}(v)-\alpha_{ilg}\right)]
\end{aligned}
$$

Then

$$
\begin{aligned}
&[\boldsymbol{\eta}_k \mid \mathbf{X}_{lg}(v),\mathbf{Y}_{lg}(v),\mathbf{Z}_{lg}(v),\mathbf{D},\mathbf{W},\overline{\boldsymbol{\eta}}_k\ ] \\
\propto & \mathrm{N}\left((\boldsymbol{\Sigma}_k^{-1}+\boldsymbol{\Omega}_{\boldsymbol{\eta}_k})^{-1}\mathbf{P}_{\boldsymbol{\eta}_k},(\boldsymbol{\Sigma}_k^{-1}+\boldsymbol{\Omega}_{\boldsymbol{\eta}_k})^{-1}\right)
\end{aligned}
$$

$$
\begin{aligned}
(15)\ &[\boldsymbol{\Sigma}_k \mid \mathbf{X}_{lg}(v),\mathbf{Y}_{lg}(v),\mathbf{Z}_{lg}(v),\mathbf{D},\mathbf{W},\overline{\boldsymbol{\Sigma}}_k\ ] \\
\propto & \mathrm{InvW}\left(\boldsymbol{\Lambda}+\boldsymbol{\eta}_k\boldsymbol{\eta}_k^T,\nu+1\right)
\end{aligned}
$$

3. Derivation of voxel-level LOOCV predictive probability Let $\mathbf{B}_i(\bar{v})$ denote all the imaging data in the brain except voxel v. First, we write out the posterior predictive

probability:

$$
\begin{aligned}
&P(D_i = k \mid \mathbf{B}_i(v), \mathbf{A}_{-i}) \\
&= \int P(D_i = k \mid \mathbf{B}_i(v), \boldsymbol{\theta}) P(\boldsymbol{\theta} \mid \mathbf{B}_i(v), \mathbf{A}_{-i}) d\boldsymbol{\theta} \\
&= \int P(D_i = k \mid \mathbf{B}_i(v), \boldsymbol{\theta}) \frac{P(\boldsymbol{\theta} \mid \mathbf{B}_i(v), \mathbf{B}_i(\bar{v}), \mathbf{A}_{-i})}{P(\boldsymbol{\theta} \mid \mathbf{B}_i(v), \mathbf{B}_i(\bar{v}), D_i = d_i, \mathbf{A}_{-i})} P(\boldsymbol{\theta} \mid \mathbf{B}_i(v), \mathbf{B}_i(\bar{v}), D_i = d_i, \mathbf{A}_{-i}) d\boldsymbol{\theta},
\end{aligned}
$$

in which

$$
\frac{P(\boldsymbol{\theta} \mid \mathbf{B}_i(v), \mathbf{B}_i(\bar{v}), \mathbf{A}_{-i})}{P(\boldsymbol{\theta} \mid \mathbf{B}_i, \mathbf{B}_i(\bar{v}), D_i = d_i, \mathbf{A}_{-i})} = \frac{P(\boldsymbol{\theta}, \mathbf{B}_i, \mathbf{A}_{-i}) P(\mathbf{B}_i, D_i = d_i, \mathbf{A}_{-i})}{P(\mathbf{B}_i, \mathbf{A}_{-i}) P(\boldsymbol{\theta}, \mathbf{B}_i, D_i = d_i, \mathbf{A}_{-i})} = \frac{P(D_i = d_i \mid \mathbf{B}_i, \mathbf{A}_{-i})}{P(D_i = d_i \mid \mathbf{B}_i, \boldsymbol{\theta})}.
$$

Thus,

$$
\begin{aligned}
\frac{P(D_i = k \mid \mathbf{B}_i(v), \mathbf{A}_{-i})}{P(D_i = d_i \mid \mathbf{B}_i, \mathbf{A}_{-i})} &= \int \frac{P(D_i = k \mid \mathbf{B}_i(v), \boldsymbol{\theta})}{P(D_i = d_i \mid \mathbf{B}_i, \boldsymbol{\theta})} P(\boldsymbol{\theta} \mid \mathbf{B}_i, D_i = d_i, \mathbf{A}_{-i}) d\boldsymbol{\theta} \\
&= \int \frac{P(\mathbf{B}_i(v) \mid D_i = k, \boldsymbol{\theta}) P(D_i = k) / P(\mathbf{B}_i(v) \mid \boldsymbol{\theta})}{P(\mathbf{B}_i \mid D_i = d_i, \boldsymbol{\theta}) P(D_i = d_i) / P(\mathbf{B}_i \mid \boldsymbol{\theta})} P(\boldsymbol{\theta} \mid \mathbf{B}_i, D_i = d_i, \mathbf{A}_{-i}) d\boldsymbol{\theta} \\
&:= \frac{P(D_i = k)}{P(D_i = d_i)} Q_{kd_i},
\end{aligned}
$$

which leads to (12).

4. Trace plots

Figure S1: Trace plots for selected voxel-level, subregion-level, and region-level parameters from posterior sampling.

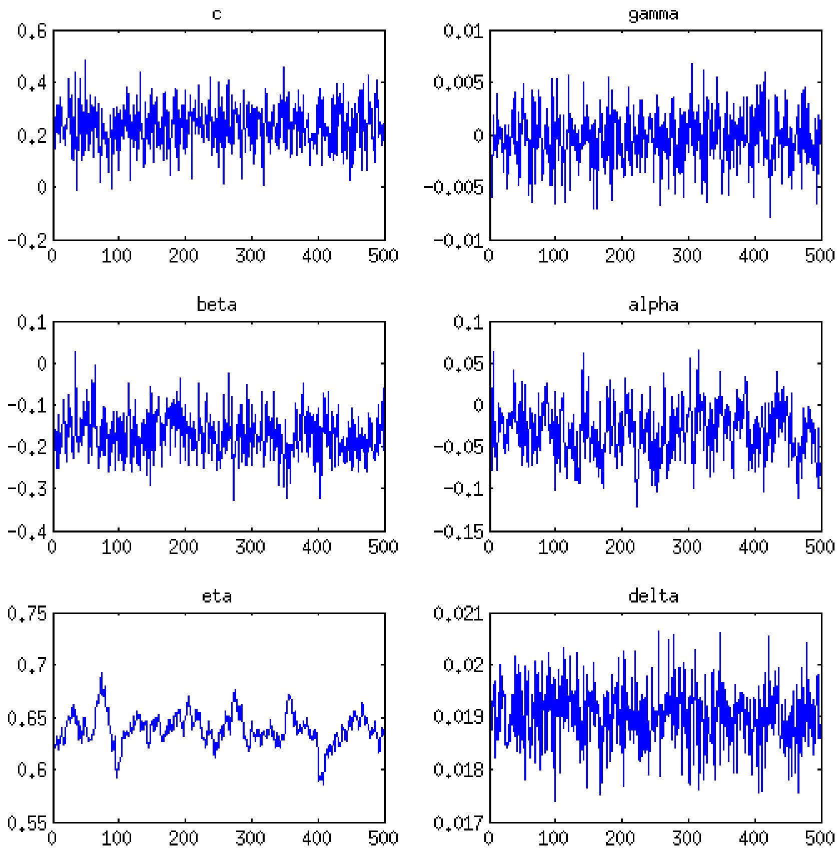

Supplement: Supplementary file 1 [file DataSheet1.pdf]
